# A comparative analysis of punching in boxing and sanda: kinematic differences based on the cross and uppercut

**DOI:** 10.3389/fspor.2024.1441470

**Published:** 2024-09-27

**Authors:** QingLou Xu, Ruiqiu Mao, Changjin Xi

**Affiliations:** ^1^ Department of Physical Education, Zhejiang Guangsha Vocational and Technical University of Construction, Jinhua, Zhejiang, China; ^2^ Graduate Department, Shenyang Sport University, Shenyang, Liaoning, China

**Keywords:** combat sports, boxing, biomechanics, multivariate regression analysis, sanda

## Abstract

**Background:**

This research aims to compare the differences in kinematic parameters associated with cross and uppercut punches between Sanda athletes (SA) and Boxing athletes (BA) to analyze their impacts on peak punching speed.

**Methods:**

The punches of BA (*n* = 20) and SA (*n* = 20) were compared utilizing a three-dimensional (3D) framework and high-speed cameras in terms of 13 key parameters. An independent samples *t*-test (*α* = 0.05) was employed to analyze the differences in punching between BA and SA. Meanwhile, a stepwise multiple linear regression equation was developed to analyze the influence of selected parameters on peak punching speed.

**Results:**

The results reveal that, among the 13 kinematic parameters, the six cross-related parameters and four uppercut-related parameters are significantly different (both *p* ≤ 0.05). The results of multivariate regression analysis unveils that the peak punching speed for the cross are influenced by the anteroposterior position of the center of gravity (in BA) and the maximum angular velocity of the shoulder (in SA). In contrast, for both BA and SA, the maximum angular velocity of the shoulder plays a critical impact on uppercut.

**Conclusions:**

These findings indicate that trunk and upper limbs significantly influence the peak punching speed, which provides suggestions for daily training regimen of SA and BA as well as their coaches.

## 1 Introduction

Boxing and Sanda are combat sports that place a premium on both technical prowess and physical strength (1). During a match, athletes typically engage in a series of continuous punches, intricate footwork, and defensive maneuvers, to score points within 3-minute rounds. The fundamental techniques in Boxing and Sanda include straight punches, swing punches, and hook punches, which are further categorized into rear hand or lead hand punches based on the tactical scenario. The rear hand, locates at the farthest position from the target, is renowned for delivering substantial force, while the lead hand occupying the closest position to the target can achieve maximum speed. Moreover, these techniques can be further distinguished into inside punches (rear hook and uppercut) and outside punches (jab and cross). The former earns its designation due to high efficacy at shorter distances within the opponent's punching range. In contrast, the straight punches like the jab/cross may not necessarily be inside the opponent's range, so they are categorized as outside punches. In a scenario where two athletes maintain a typical non-attacking distance (about 1–1.5 meters), they often use jab because it is likely outside the opponent's range and can set up subsequent strategic opportunities. Recognizing these nuances in punching techniques is conductive to highlighting the importance of analyzing the kinematic performance of inside and outside punches, and then to enhance the understanding of athletes and coaches alike. Given that the Sanda was introduced at the 2008 Beijing Olympics and the number of participation in the Sanda in the sport worldwide is significantly increasing, it is crucial to investigate and understand the possible biomechanical differences between Sanda and Boxing.

Although numerous studies have delved into the punching performance, most of them were limited in biomechanical analysis (2, 3), so that it is hard to establish substantial assistance to athletes through interconnections among various quantitative data. Furthermore, most of these studies independently focus on male and female athletes and fail to reveal the potential differences in combat sports. This scarcity of empirical evidence poses a challenge for coaches and athletes to fully understand the approaches for enhancing punching performance based on kinematic analysis and to quantitatively assess the knowledge and information impacting the punching performance (2). In line with previous assessments of athletic techniques (4, 5), collecting kinematic data of the cross and uppercut and analyzing their impact on maximum punching speed can offer valuable insights into the complexity of inside and outside punch techniques. This, furthermore, assists in developing targeted intervention training specifically tailored for the cross and uppercut. In this research, a refined and efficient method was proposed to explore the differences in peak punching speed between Boxing athletes (BA) and Sanda athletes (SA), and a stepwise multiple linear regression equation was established to assess the impact of kinematic parameters on peak punching speed.

In summary, this research is developed to compare the kinematic performance of cross and uppercut punches between BA and SA using the biomechanical theory and to analyze the impact of kinematic parameters (independent variables) on punching speed (a dependent variable) through establishing a stepwise multiple linear regression equation.

The hypotheses in this research include (A) There are significant differences between BA and SA in kinematic performance (velocity parameters, center of gravity parameters, or angle parameters) in cross and uppercut; and (B) The center of gravity and upper limbs are determining factors of the maximum punching speed in crosses and uppercuts.

## 2 Materials and methods

### 2.1 Participants

According to the G*power sample size estimation software, Power = 0.8 and α = 0.05 were set in two groups, the total sample size was estimated to be 34, with 17 participants in each group. Actually, 20 BA and 20 SA at Shenyang Sport University were recruited. The general data of BA are expressed in the form of mean ± standard deviation (M ± SD) as 19.41 ± 0.69 years old, 173.2 ± 7.4 cm, 64.7 ± 10.9 kg, and 6.26 ± 1.35 years of training, while those of the 20 SA are 19.38 ± 0.76 years old, 169.5 ± 7.1 cm, 59.8 ± 11.1 kg, and 5.72 ± 1.18 years of training. As the years of training showed, these participants have been immersed in comprehensive professional Boxing or Sanda training or coursework since their high school years or even earlier, boasting a track record of involvement in no fewer than 10 formal amateur or student matches, so they are proficient in cross and uppercut techniques. Prior to the experiment, their physical conditions were assessed to ensure they experienced no significant injuries in the past 6 months and no recent high-intensity training. Additionally, stringent measures were implemented to confirm they refrained from high-intensity training in the 24 h leading up to the tests, coupled with an ample rest period exceeding 8 h. This experiment was conducted in compliance with the *Declaration of Helsinki*, has obtained the written informed consents from all participants, and approved by the Ethics Committee of Shenyang Sport University [Ethics (2024) No. 12].

### 2.2 Experimental procedure

**Figure 1:**
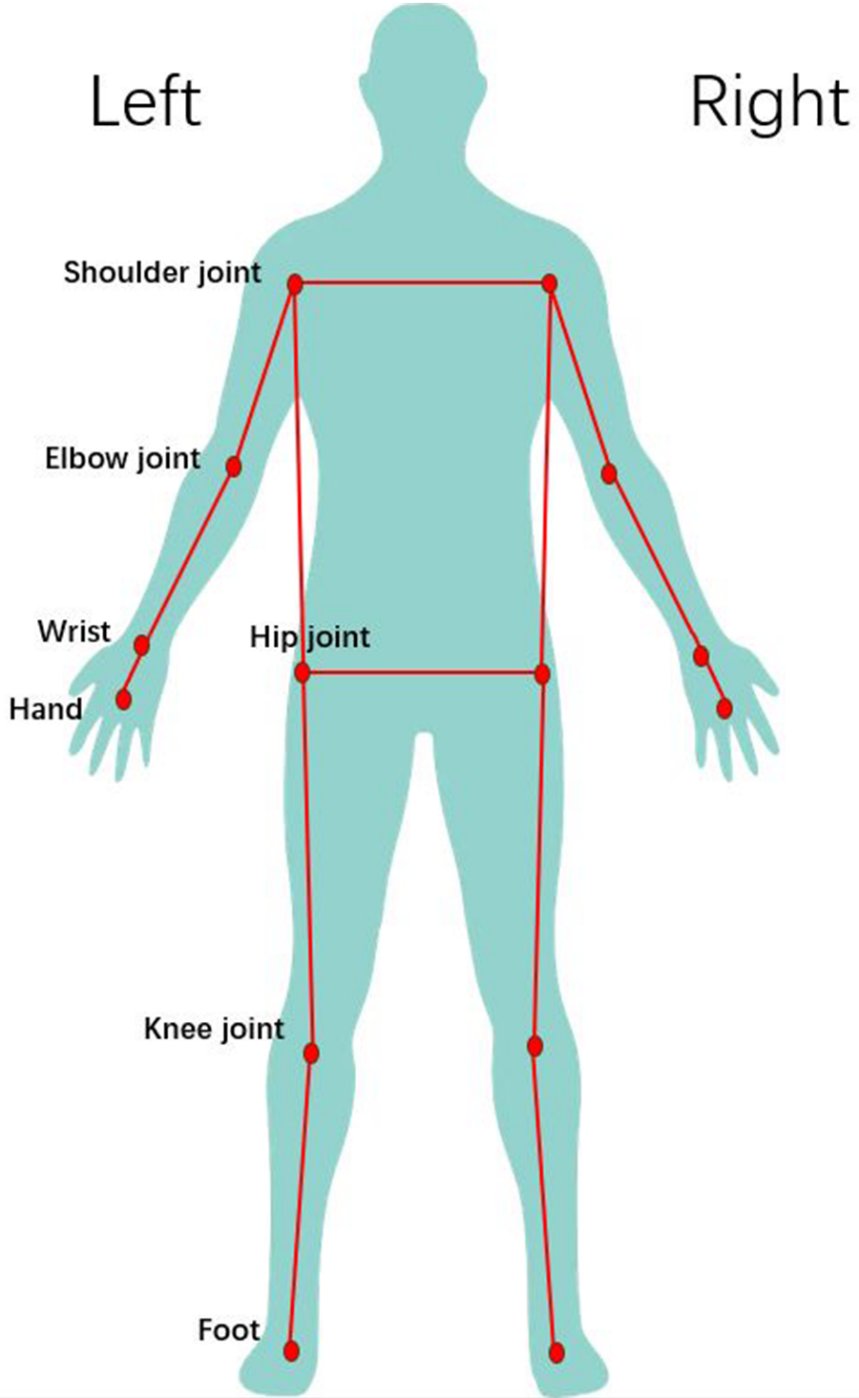
Body joint points.

Two high-speed cameras (SONY HVR-V1C, manufactured in Japan) were employed to capture the punching dynamics of athletes in a fixed position. The cameras were positioned directly in front and to the right of the athletes, with a rapid shooting frequency of 200 Hz. Before and after the recordings, a 3D framework (013-c) was adopted for spatial position calibration (Figure 1). The calibrated 3D human motion analysis system adopted in this research was guaranteed with a high accuracy, with a relative error of less than 2% (6). The experimental procedures unfolded at the Boxing training facility of Shenyang Sport University, with a steadfast commitment to upholding the principles outlined in the *Declaration of Helsinki*. Prior to the commencement of the tests, athletes underwent thorough warm-up sessions, and the experimental procedures were diligently demonstrated to ensure a clear understanding. To ensure a more rigorous analysis of technical movements, all athletes were specifically instructed to use an orthodox (right-arm) stance during the punching. Throughout the tests, athletes maintained the positions calibrated by the 3D framework and executed three air punches for both the cross and uppercut with maximum force (without hitting a target). Notably, each punch originated from a standardized defensive posture, so that every successive punch can be continuously self-adjusted.

**Figure 2:**
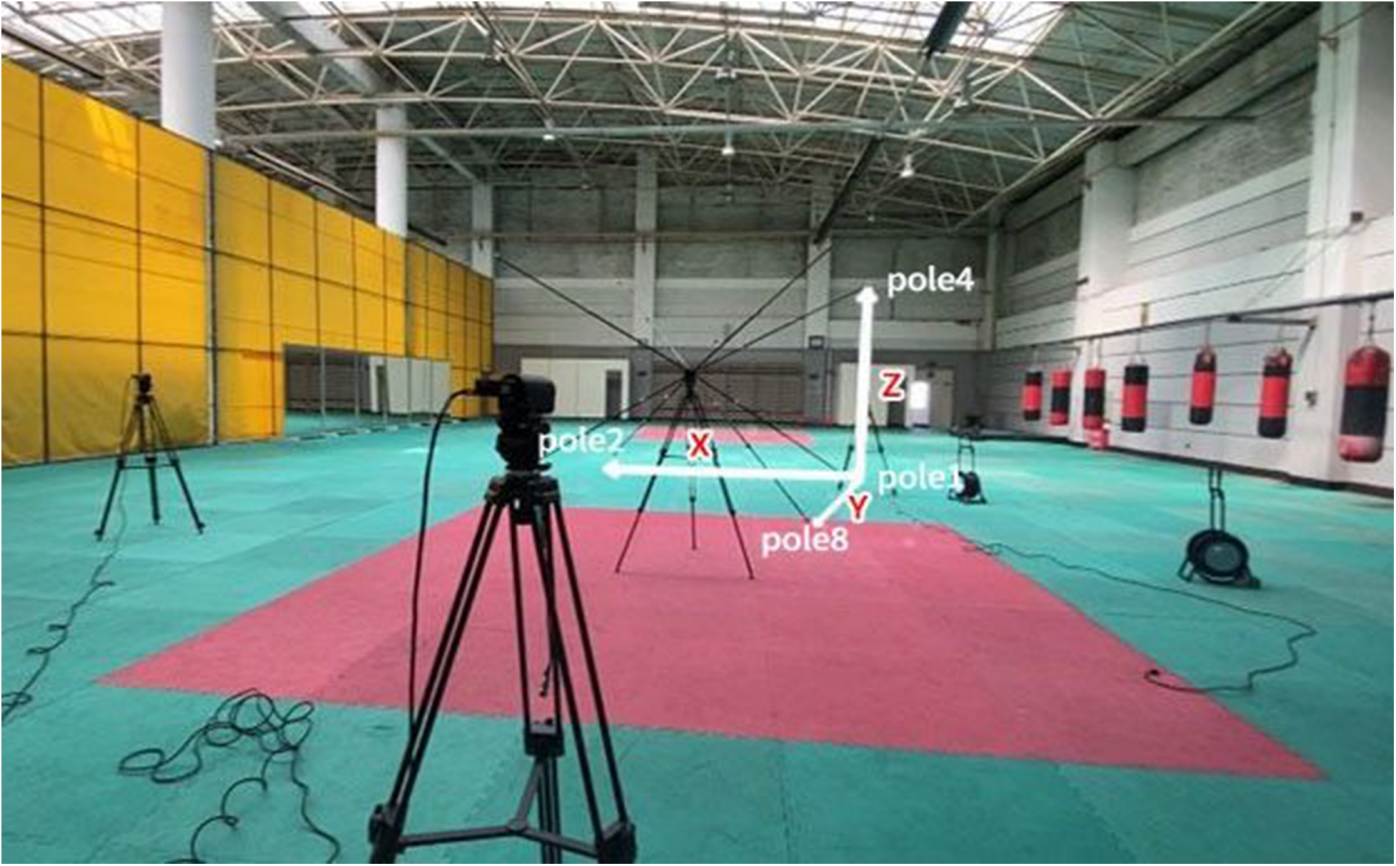
The high-speed cameras and three-dimensional framework.

**Figure 3:**
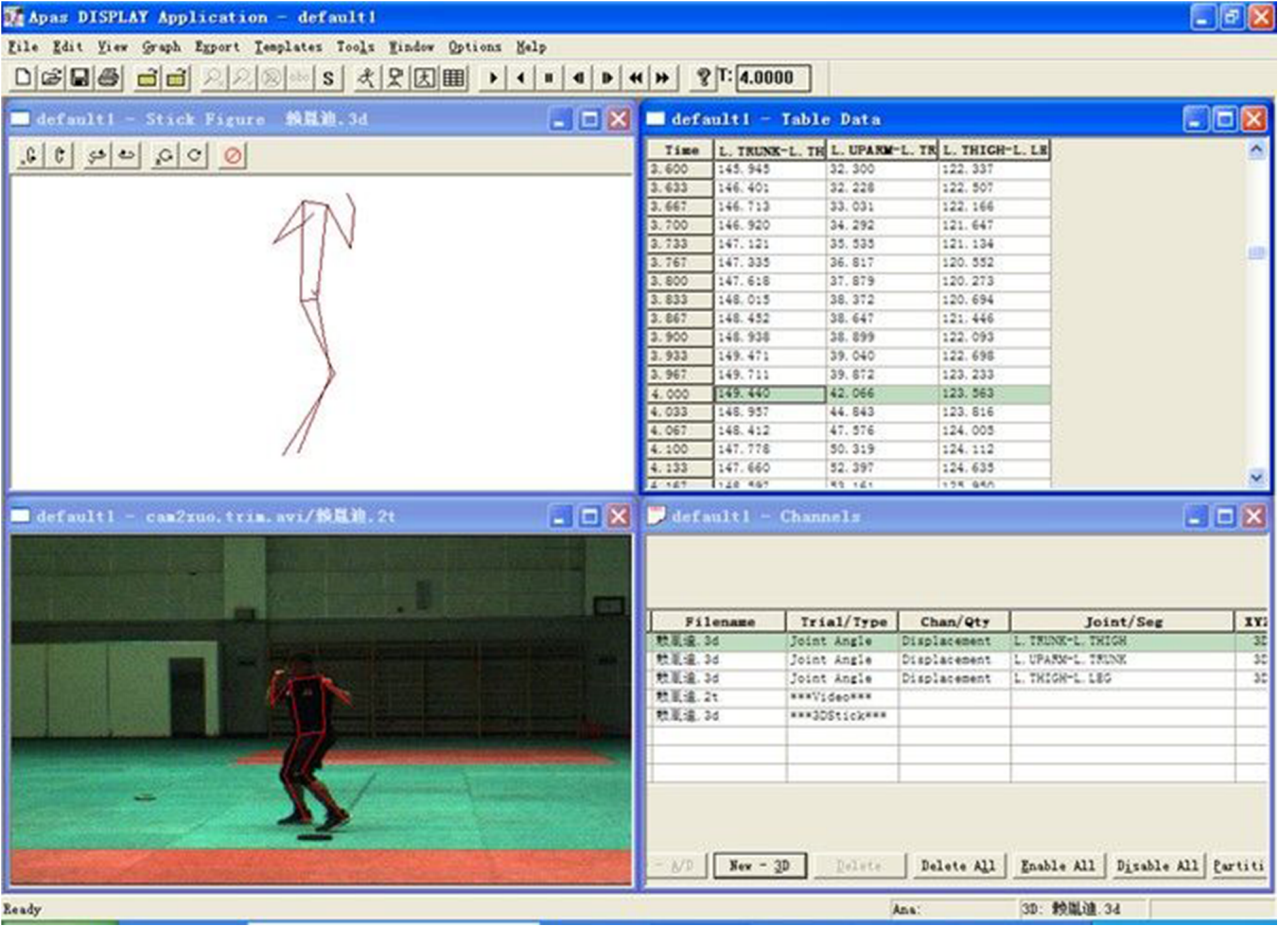
Video motion analysis setup.

After capturing the footage, the obtained videos were analyzed using the ariel performance analysis system (APAS, USA). Specifically, 14 anatomical points on the body were selected for analysis (Figures 2, 3), and a low-pass filtering method (with a frequency of 10 Hz) was applied for smoothing. The punching process involves the sequential rotation of the torso, propelling the upper arm and forearm in the punching direction, so the punching action conforms to the principles of whip-like motion in the upper limb. The posture assumed during the punching greatly determines the position of the center of mass related to the supporting surface, and dynamic changes in the center of mass are crucial for stability and flexibility of athletes. Joint angles during the punching process not only mirror the standardization of movement technique but also determine the effectiveness of hitting the target. In this research the kinematic performance of cross and uppercut among BA and SA were compared, and some parameters were examined, including the speed parameters, center of mass parameters, and angle parameters. During the punching, all joint angles were taken from the body joints on the right side, and all angular velocities were made from the horizontal plane/*X*-axis of the human body. The selected numerical values formed seven speed parameters, three angle parameters, and three center of mass parameters, as listed in Table 1 (7).

**Table 1:** Various kinematic parameters.

	Parameters	Explanation
Velocity	Hand _*ν*	Maximum punching speed refers to the line speed of the hand
	Shoulder _*ω*	Maximum shoulder angular velocity refers to the change in the angle of projection of the angle formed by the line joining the two shoulders on the horizontal plane per unit time
	Trunk _ω	Maximum torso rotation angular velocity refers to the change in torso rotation angle per unit time
	Waist _ω	Maximum hip angular velocity refers to the change in the angle of projection of the angle formed by the line joining the two hips on the horizontal plane per unit of time
	Elbow_ω	Maximum elbow flexion and extension angular velocity refers to the change in the angle between the upper arm and the forearm per unit of time
	Shoulder joint_ω	Maximum shoulder joint angular velocity refers to the change in the angle between the upper arm and the human torso per unit of time
Center of mass	Gravity _X	Range of movement of the body's center of gravity along the *X*-axis
	Gravity _Y	Range of movement of the body's center of gravity along the *Y*-axis
	Gravity _Z	Range of movement of the body's center of gravity along the Z-axis
Angular	Trunk _*θ*	Maximum torso rotation angle refers to the angle between the shoulder joints and the hip joints projected on the horizontal plane
	Shoulder joint_θ	Shoulder joint angle of the maximum punching speed
	Hip_θ	Hip joint angle of the maximum punching speed
	Knee_θ	Knee joint angle of the maximum punching speed

All joint angles were taken from the body joint on the right side during punching, and all angular velocities were taken from the horizontal plane/*X*-axis of the human body.

### 2.3 Statistical analysis

All kinematic data were descriptively statistically analyzed, expressed as M ± SD and Kolmogorov-Smirnov (KS) normality tests (*p *≤ 0.05). Subsequently, an independent samples *t*-test (α = 0.05) was employed to analyze the differences in punching between BA and SA. Finally, SPSS 25 was utilized for stepwise multiple regression analysis to establish a comprehensive multiple linear regression equation that identifies the primary factors influencing the dependent variable (hand _v).

This method introduced all independent variables (12 in total) at once and tested and eliminated the variables that lost statistical significance to address the multicollinearity. Given the assumptions for establishing the multiple regression equation model, the Durbin-Watson (DW) value was reported for validation. After establishment, the multiple regression model was further corrected and tested. Ultimately, six optimal regression equations were provided, including the adjusted R^2^, standard estimation error, F-value, Sig, DW value, and Beta value, for each equation.

## 3 Results

Tables 2, 3 present the kinematic performance. Tables 4–7 summarize the results of a multiple regression analysis.

**Table 2:** Comparison of kinematic performance on cross.

	SA(*n = 20*)	BA(*n = 20*)	*t*	*p*	*d*
Hand _ν(*m/s*)	8.48 ± 0.61	7.30 ± 0.49	*1.016*	*<0.01*	2.132
Shoulder _ω(*rad/s*)	12.95 ± 1.12	11.55 ± 1.23	*1.079*	*<0.01*	1.190
Waist _ω(*rad/s*)	10.47 ± 1.17	10.40 ± 1.10	*0.197*	*0.844*	0.062
Trunk _ω(*rad/s*)	10.83 ± 1.63	11.64 ± 1.92	*−1.433*	*0.144*	−0.455
Elbow_ω(*rad/s*)	11.57 ± 1.85	10.44 ± 2.82	*1.494*	*0.050*	0.474
Sh_joint_ω(*rad/s*)	12.78 ± 4.70	11.48 ± 1.00	*1.205*	*0.254*	0.382
Gravity _X(*mm*)	117.90 ± 13.41	118.73 ± 42.52	*−0.084*	*0.934*	−0.026
Gravity _Y(*mm*)	85.56 ± 6.95	55.60 ± 20.20	*6.271*	*<0.01*	1.983
Gravity _Z(*mm*)	43.39 ± 5.51	47.37 ± 9.19	*−1.580*	*0.127*	−0.525
Trunk _θ(*°*)	0.81 ± 0.27	0.92 ± 0.29	*−1.223*	*0.227*	−0.393
Sho_joint_θ(*°*)	105.29 ± 1.44	105.53 ± 2.68	*−0.346*	*0.707*	−0.112
Hip_θ(*°*)	158.22 ± 1.26	156.97 ± 1.38	*−1.804*	*<0.01*	0.946
Knee_θ(*°*)	176.48 ± 0.71	168.49 ± 1.20	*−0.31*	*<0.01*	8.104

Italic values means *p* ≤ 0.05.

**Table 3:** Comparison of kinematic performance on uppercut.

	SA(*n = 20*)	BA(*n = 20*)	*t*	*p*	*d*
Hand _ν(*m/s*)	11.00 ± 1.05	9.88 ± 1.11	*0.370*	*<0.01*	1.037
Shoulder _ω(*rad/s*)	12.88 ± 2.10	11.61 ± 3.04	*1.543*	*0.131*	0.486
Waist _ω(*rad/s*)	10.30 ± 1.65	12.20 ± 1.62	*−3.690*	*<0.01*	−1.162
Trunk _ω(*rad/s*)	8.89 ± 1.10	7.76 ± 1.54	*2.657*	*0.016*	0.844
Elbow_ω(*rad/s*)	6.37 ± 2.59	5.74 ± 2.92	*0.725*	*0.328*	0.228
Sh_joint_ω(*rad/s*)	10.92 ± 2.96	7.14 ± 4.43	*3.176*	*<0.01*	1.003
Gravity _X(*mm*)	88.76 ± 40.23	95.56 ± 41.49	*−0.526*	*0.566*	−0.166
Gravity _Y(*mm*)	90.26 ± 18.26	86.21 ± 23.26	*0.612*	*0.542*	0.194
Gravity _Z(*mm*)	56.94 ± 14.05	54.11 ± 11.62	*0.693*	*0.531*	0.219
Trunk _θ(*°*)	0.94 ± 0.22	0.86 ± 0.44	*0.729*	*0.479*	0.229
Sho_joint_θ(*°*)	65.32 ± 2.38	64.21 ± 3.22	*0.982*	*0.158*	0.392
Hip_θ(*°*)	158.39 ± 1.12	158.32 ± 0.96	*0.218*	*0.826*	0.067
Knee_θ(*°*)	152.65 ± 1.37	152.29 ± 1.65	*0.754*	*0.430*	0.237

Italic values means *p* ≤ 0.05.

**Table 4:** Ba's model abstract of peak punching speed on cross.

Model	Estimated standard error	Adjusted R^2^	*F*	*Sig.*	Durbin-Watson
1	0.43933	0.433	15.511	0.001	2.2
2	0.34653	0.647	11.931	0.003	
3	0.19167	0.892	39.57	0.000	
4	0.14904	0.935	11.461	0.004	

Dependent variable: hand_ν. Predictive value a: elbow_ω. Predictive value b: elbow_ω; gravity _Y. Predictive value c: elbow_ω;gravity _Y; trunk_ω. Predictive value d: elbow_ω;gravity _Y; trunk_ω; shoulder joint_ω. Beta: elbow_ω = 0.646, gravity _Y = 0.725, trunk_ω = 0.507, shoulder joint_ω = 0.219.

**Table 5:** Sa's model abstract of peak punching speed on cross.

Model	Estimated standard error	Adjusted R^2^	*F*	*Sig.*	Durbin-Watson
1	0.27421	0.688	42.98	0.000	2.199
2	0.22652	0.787	9.377	0.007	
3	0.20158	0.832	5.468	0.033	

Dependent variable: hand_ν. Predictive value a: elbow_ω. Predictive value b: elbow_ω; shoulder_ω. Predictive value c: elbow_ω; shoulder_ω;trunk_ω. Beta: elbow_ω = 0.376, shoulder_ω = 0.406, trunk_ω = 0.294.

**Table 6:** Ba's model abstract of peak punching speed on uppercut.

Model	Estimated standard error	Adjusted R^2^	*F*	*Sig.*	Durbin-Watson
1	0.71374	0.542	23.493	0.000	1.859
2	0.54346	0.735	14.047	0.002	
3	0.49006	0.784	4.906	0.042	

Dependent variable: hand_ν. Predictive value a: shoulder_ω. Predictive value b: shoulder_ω; gravity _Y. Predictive value c: shoulder_ω; gravity _Y; gravity _Z. Beta:shoulder_ω = 0.427, gravity _Y = 0.352, gravity _Z = 0.313.

**Table 7:** Sa's model abstract of peak punching speed on uppercut.

Model	Estimated standard error	Adjusted R^2^	*F*	*Sig.*	Durbin-Watson
1	0.7157	0.587	27.965	0.000	1.950
2	0.6169	0.693	7.232	0.016	

Dependent variable: hand_ν. Predictive value a: shoulder_ω. Predictive value b: shoulder_ω; gravity _Y. Beta: shoulder_ω = 0.563, gravity _Y = 0.405.

### 3.1 Kinematic performance

As revealed in Tables 2, 3, the peak punching speed of the cross of the BA is 16.2% higher than that of SA (*p *≤ 0.05), showing a significant difference. Significant differences are observed in maximum angular velocity of the shoulder (*p *≤ 0.05) and maximum elbow flexion and extension angular velocity (*p *≤ 0.05), while those in the other three angular velocities are not remarkable. Notably, BA have the highest angular velocity in the maximum angular velocity of the shoulder, while SA possess the highest angular velocity in the maximum trunk rotation angular velocity. The lowest angular velocity is observed for both BA and SA during the cross in the maximum angular velocity of the hip. In terms of center of gravity parameters, the three types of movements associated with the cross demonstrate a significant difference solely in the *Y*-axis range (*p *≤ 0.05). The average *Y*-axis range for cross of BA surpasses that of the SA by 53.8%. Regarding angle parameters, differences are not significant in the maximum trunk rotation angle and shoulder joint angle during punching. The knee joint angle (*p *≤ 0.05) and hip joint angle (*p *≤ 0.05) are greatly different, with BA having a 4.7% greater knee joint angle than SA.

There exist substantial differences in the peak punching speed of the uppercut between BA and SA (*p *≤ 0.05), with 11.3% higher for the BA. Among all the angular velocity parameters, only the maximum angular velocity of the hip, maximum torso rotation angular velocity and the maximum angular velocity of the shoulder are greatly different (*p *≤ 0.05). The maximum angular velocity of the shoulder achieves the highest angular velocity parameter for BA, while that for SA is the maximum angular velocity of the hip. Furthermore, the lowest angular velocity parameter for both BA and SA is the maximum elbow joint angular velocity. Moreover, there are no considerable differences in all seven parameters related to center of gravity and angle parameters.

### 3.2 Regression analysis

#### 3.2.1 Cross

To temporarily circumvent the covariance, stepwise analysis can be employed to search the optimal combination of independent variables, and the most highly associated independent variables are automatically selected to enter into the model. As shown in Table 4, there are four independent variables in influence model of cross of BA, including elbow_*ω*(*rad/s*), gravity _X(*mm*), trunk_*ω*(*rad/s*) and shoulder joint_*ω*(*rad/s*), which can explain 94.8% of the variance or 93.5% after adjustment. The statistical significance of this explanatory power, encompassing four independent variables, can be determined in the final equation on the basis of the F-test results [*F*(*3,16*)* = 69.044, p < 0.001*]. The equation is as follows:

Y = 0.382 + 0.646*elbow_*ω(rad/s) *+ 0.725*gravity _Y*(mm) *+ 0.507*trunk_*ω(rad/s)*- 0.219*shoulder joint_*ω(rad/s)*.

This equation was used for actual performance prediction, and the estimated standard error was 0.149.The coefficient estimation of the stepwise analysis (Bate) revealed that the order of factors influencing the peak punching speed of BA in cross is: gravity _Y(*mm*)>elbow_*ω* (*rad/s*)>trunk_*ω*(*rad/s*)>shoulder joint_*ω(rad/s)*.

Similarly, the results of the peak punching speed influence model of SA in cross are shown in Table 5, and the equation includes three independent variables, as follows (Estimated standard error=0.202):

Y = 4.706 + 0.376*elbow_*ω*(*rad/s*) + 0.406*shoulder_*ω*(*rad/s*) + 0.294*trunk_*ω*(*rad/s)*.

The coefficient estimation of the stepwise analysis (Bate) reveals that the order of factors influencing the maximum punching speed of SA in cross is:shoulder_*ω(rad/s)*>elbow_*ω (rad/s)*>trunk_*ω*(*rad/s*).

#### 3.2.2 Uppercut

The results of the peak punching speed influence model of BA in uppercut are shown in Table 6, and the equation as follows (Estimated standard error=0.496):

Y = 0.604 + 0.427*shoulder_*ω(rad/s*) + 0.352*gravity _Y(*mm*) +0.313*gravity _Z*(mm)*.

Factors influencing the maximum punching speed of BA in uppercut can be sequenced as follows: shoulder_*ω*(*rad/s*)>gravity _Y(*mm*)>gravity _Z(*mm*).

The results of the peak punching speed influence model of SA in uppercut are shown in Table 7, and the equation as follows (Estimated standard error=0.619): Y = 6.684 + 0.405* gravity _Y(*mm*) + 0.563* shoulder_*ω (rad/s*).

The order of factors influencing the maximum punching speed of SA in uppercut was: shoulder _*ω* (*rad/s)*>gravity _Y(*mm).*

## 4 Discussion

### 4.1 Cross

Cross is categorized as an outside punch with a long reach and is frequently applied in matches despite having a lower hit rate. It serves as a primary method for scoring at mid-range and usually employed for counterattacks or as part of a combination of punches. As a result, it is of strategic significance in various match scenarios for BA and SA.

As depicted in Table 2, the peak punching speed for BA in cross is 8.48 ± 0.61 m/s while 7.30 ± 0.49 m/s for SA, which align with the findings of Piorkowski (8.22 ± 0.68 m/s) (3) and Kimm (7.70 ± 1.50 m/s) (8), respectively. Notably, BA have higher cross speed parameters in all aspects except for the maximum trunk rotation angular velocity. The maximum trunk rotation speed is crucially determined by the linear speed of hip and shoulder movements. Studies analyzing surface electromyographic data have demonstrated that the peak speed sequence for cross is ‘hip-shoulder-elbow-wrist-hand’ (9). Throughout the execution of the cross, the elbow joint rapidly extends forward under the influence of the shoulder joint. This action reduces the rotational inertia of the upper limb around the longitudinal axis and increases the angular velocity of forearm internal rotation, thus facilitating the accumulation and utilization of elastic energy of the elbow joint muscle group. Therefore, a faster trunk rotation speed contributes to utilizing the energy from the lower limb segment and increasing the energy transfer from the proximal trunk segment to the distal shoulder joint segment. However, contrary studies have pointed out that the athletes, during the cross, exhibit a lower flexion-extension ratio of the knee joint and lower internal-external rotation ratio of the shoulder joint. This indicates that as angular velocity increases, there is a significant decrease in the flexor muscle strength of the knee joint and the internal-external rotation strength of the shoulder joint in athletes. Consequently, the flexion-extension and internal-external rotation ratios decrease notably, compromising the joint stability and elevating the risk of muscle strain among athletes. Therefore, the greater maximum trunk rotation angular velocity during cross of SA is inferred to propel the speed of other upper limb joints, contributing to an increased end punch speed. Moreover, this results in a greater maximum trunk rotation (0.92 ± 0.29°) compared to BA. This is beneficial for utilizing the energy from the lower limb segment and highlights the trunk rotation decreases shoulder joint stability during punching, thereby increasing the risk of injury. Additionally, it necessitates increased mobilization of trunk muscles, thus reducing the punch quality. As a result, it is recommended that athletes prioritize strength training during punching exercises, with an emphasis on explosive power training and supplementary maximum strength training. This approach aims to optimize the efficiency of strength quality transfer to punching speed and reduce the risk of injury (10).

The center of gravity movement range on the Y-axis of BA is greater than that of SA, indicating a more pronounced forward center of gravity. This forward displacement during punching, along with the transfer of more body mass forward, enhances the mass of proximal segments and increases the rotational inertia of trunk, thereby increasing the flexibility of the cross and accelerating its speed. However, this advantage comes at the expense of reduced stability on the *Y*-axis during punching and a longer path for retracting the punch. It is essential for athletes to exercise with caution when the cross is used during matches, as it can be easily exploited by the opponent, leading to a loss of initiative (11). Various studies utilizing diverse methods of center of gravity measurement have consistently proven that winners in combat sports show greater variation in their center of gravity position compared to losers (12). Punching standards (skill level) are positively correlated with external load (frequency of offensive and defensive actions). Thus, winning outcomes are associated with a high offensive frequency and a low defensive frequency. In this context, the motion range of the center of gravity is not only crucial for optimizing punching techniques but also for the overall outcome of combat sports like Boxing and Sanda.

The primary distinction in angle parameters is observed in the knee joint, which is a critical factor for effectively transferring leg thrust that subsequently propels force to the upper limbs. The push-off from the back leg also aids in initiating body movement during punching. Studies have demonstrated that, at the same angular velocity, the correlation coefficient of punching force between the front and rear hand straight punches and the knee joint surpasses that of the shoulder joint (10). Furthermore, an active leg push-off during the cross exerts a positive impact on punching speed (13). Notably, the knee joint angle of the same-side lower limb is greater in BA than in SA during the cross. It signifies a more substantial push-off, which enables a more efficient transfer of force upwards, thus contributing to increased punching power and speed. Additionally, a greater hip joint angle in BA results in an overall trunk rotation towards the *Y*-axis and shift of the body's center of gravity further forward, thus facilitating the effective transfer of energy from proximal to distal segments and enhancing punching speed. In addition, it contributes to the observed discrepancy in cross speed between BA and SA, signifying that, apart from differences in upper limb strength, lower limb strength plays a decisive role.

The outcomes of regression analysis results (Tables 4, 5) reveal that the factor influencing the maximum punching speed of BA in cross (gravity_Y) is difference from that affecting SA in cross (shoulder_ω). For SA, range of anteroposterior movement of the center of gravity is the primary factor affecting the maximum punching speed. Actually, it not only influences punching speed but also crucial in determining its stability and flexibility on the *Y*-axis. Relevant studies suggest that electromyographic characteristics of the cross movement involve early activation of the calf muscles and biceps femoris of the lower limb (14), with the anterior deltoid exhibiting the highest percentage of activation. Additionally (9), the integrated electromyographic value of the upper limb muscle group is greater than that of each muscle group in the lower limbs (15), suggesting a relatively higher muscle activation level in the upper limbs during the cross. Moreover, Daniel Dinu pointed out the significant contribution of the elbow joint in the upper body to the cross (16). From this perspective, the result indicating that maximum punching speed of SA in cross is influenced by the maximum angular velocity of the shoulder appears more reasonable. Furthermore, the maximum elbow joint angular velocity and the maximum trunk rotation speed are secondary factors influencing the maximum punching speed of the cross. In this regard, the regression analysis results demonstrate a tendency toward consistency between the Boxing and Sanda. Therefore, both BA and SA are recommended to prioritize the development of upper limb strength in cross training. Besides, they should understand the positive effect of rapid shoulder rotation on the energy transfer between body segments, and improve the capacity for accumulation and utilization of elastic potential energy in the elbow joint muscle group. The characteristic speed overlay of the cross, involving sequential braking of the shoulder, elbow, and hand, necessitates the design of end-release training exercises to align with this pattern. Studies have indicated that a focused 6-week elastic resistance training regimen can effectively improve the coordination and cooperation of upper limb muscles, significantly increasing the peak speed of the cross (6%–11%; *p *< 0.01) (17). Incorporating specific strength movements such as using a 30% RM single-arm push unilateral barbell and elastic resistance punching can target the triceps and anterior deltoid, leading to substantial improvements in upper limb strength development and punching speed (18). Moreover, attention should be directed towards the transmission of total body strength during the cross and the change in the body's center of gravity position to strengthen the stability of the center of gravity in the anteroposterior direction.

### 4.2 Uppercut

The uppercut is classified as an inside punch, and it is distinguished by rapid and abrupt force generation and a short motion path. It is more complex in movement details and striking technique compared to the cross and hook, yet its striking power is equally formidable. Widely employed in close-quarters combat, the uppercut stands out as the most frequently used offensive technique, often resulting in knockouts.

As shown in Table 3, the maximum punching speed of the uppercut is greater than that of the cross, the same as the findings of Daniel Dinu (16). However, Stanley et al. pointed out that the uppercut exhibits a higher peak speed than both the cross and rearhook, introducing a contentious aspect (2). This may be attributed by potential discrepancies in the punching method (punching trajectory) and testing method (target punching vs. air punching). Furthermore, Stanley highlighted that the position of the punching arm relative to the center of mass during the uppercut made by BA might represent the optimal configuration for generating muscular torque at the shoulder joint. This observation aligns with the significant difference in the maximum shoulder joint angular velocity obtained in this research (*p *< 0.01). Uppercut involves the elbow joint moving towards the target at a nearly fixed angle, while the shoulder joint rapidly flexes and extends in the sagittal plane, followed by abduction, protraction, and adduction. This explains the significantly greater maximum shoulder joint angular velocity compared to the maximum elbow joint angular velocity for the uppercut in this research.

Since the direction of the center of gravity movement during punching aligns with that of trunk rotation, the uppercut initiates its center of gravity movement towards the *X*-axis (left-right), followed by movement along the *Y*-axis (forward-backward) and Z-axis (up-down). Comparing the center of gravity differences between BA and SA during uppercut shows a smaller range of movement on the *X*-axis but a greater range on the *Y*-axis of the BA. This suggests that BA possess greater stability on the *X*-axis in comparison to that on the *Y*-axis, simultaneously amplifying forward inertia to enhance punching speed. However, the extent of movement of the center of gravity on the Z-axis during punching is contingent upon the timing of the final braking moment of the punch. Studies have demonstrated the strong electromyographic activity in the latissimus dorsi during the uppercut (19). This actively aims to achieve a greater impulse in the final punch segment through timely braking of the upper arm and forearm using the latissimus dorsi (19). Therefore, the timing of braking determines the position of the center of gravity on the Z-axis, the angle of the shoulder joint, and the magnitude of the final impulse during the punch. To some extent, the positions of the center of gravity on the Z-axis and the angle of the shoulder joint during the punch are directly correlated with punching speed and final impulse. This elucidates why BA have greater shoulder joint angles and maximum trunk rotation angles during uppercut and higher punching speeds. Despite being the least frequently employed technique in matches, the uppercut demonstrates a higher punching speed to the cross, boasts a vertical trajectory (moving below the opponent's line of sight), unpredictability (limited use in matches), and immense impact force (20). Therefore, both coaches and athletes are encouraged to prioritize this technique and augment its application in training and competition.

Results of the multivariate regression analysis (Tables 6, 7) reveal that the shoulder_*ω* significantly impacts the peak punching speed of both BA and SA in uppercut. It, subsequently, influences the changes in center of gravity in the anteroposterior and vertical directions. Considering the characteristics of combat sports, a stance with the feet positioned front to back and the front foot turned inward is favored to mitigate exposure to counterattacks, absorb anteroposterior impact forces, and satisfy the punching and foot movement. This stance enhances the stability and flexibility on the *X*-axis and *Y*-axis while increasing the likelihood of changes in the body's center of gravity. Prior research emphasizes the importance of enhancing maximum strength and speed of force application during the active push-off phase, both front and back, to boost the punching speed of the uppercut (21). Both BA and SA are recommended to prioritize the coordination of body center of gravity movement with active leg push-off during uppercut training. Meanwhile, emphasis should be placed on incorporating strength exercises targeting the core area. Additionally, during the training of uppercut, SA should pay particular attention to effective shoulder rotation and the timing of upper limb braking to enhance energy transfer from the proximal shoulder joint to the distal segments of the upper limb. In contrast, BA should focus on maintaining the alignment of the center of gravity with the direction of the punch, amplifying the movement of body mass in the direction of the punch to enhance punching speed.

## 5 Limitations

(1) The experimental environment is quite different from the competition or real-life punching environment, and the Hawthorne effect may occur during the testing, resulting in different punching results from the usual. Consequently, subsequent studies are hoped to comparatively analyze the punching situation under competition conditions. (2) The indicators selected in this research have some limitations, such as lacking in physiological, biochemical and kinetic parameters. As a result, future studies can be more refined to provide a comprehensive understanding of the punching phenomenon. (3) Low feasibility of inter-comparison and limited sample size affect the statistical results, which could be made more rigorous by having a larger sample size or a more rigorous statistical approach to comparative analysis.

## 6 Conclusions

Remarkable differences are observed in speed, center of gravity, and angle parameters of cross and uppercut performed by Boxing athletes and Sanda athletes. Boxing athletes generally outperform Sanda athletes in multiple speed parameters, while the center of gravity and angle parameters exhibit both similarities and differences. Furthermore, results in this research reveal that the primary factors affecting punching speed are the trunk and upper limbs, with the lower limbs playing a predominant role in maintaining the overall body stability and providing appropriate push-off force for upward transmission.

## Data Availability

The original contributions presented in the study are included in the article/Supplementary Material, further inquiries can be directed to the corresponding author.
